# Evaluating Contextual Processing in Diffusion MRI: Application to Optic Radiation Reconstruction for Epilepsy Surgery

**DOI:** 10.1371/journal.pone.0101524

**Published:** 2014-07-31

**Authors:** Chantal M. W. Tax, Remco Duits, Anna Vilanova, Bart M. ter Haar Romeny, Paul Hofman, Louis Wagner, Alexander Leemans, Pauly Ossenblok

**Affiliations:** 1 Image Sciences Institute, University Medical Center Utrecht, Utrecht, The Netherlands; 2 Department of Biomedical Engineering, Biomedical Image Analysis, Eindhoven University of Technology, Eindhoven, The Netherlands; 3 Department of Mathematics and Computer Science, Eindhoven University of Technology, Eindhoven, The Netherlands; 4 Department of Electrical Engineering, Mathematics and Computer Science, Delft University of Technology, Delft, The Netherlands; 5 Department of Function and Medical Technology, Epilepsy Center Kempenhaeghe, Heeze, The Netherlands; University of Minnesota, United States of America

## Abstract

Diffusion MRI and tractography allow for investigation of the architectural configuration of white matter in vivo, offering new avenues for applications like presurgical planning. Despite the promising outlook, there are many pitfalls that complicate its use for (clinical) application. Amongst these are inaccuracies in the geometry of the diffusion profiles on which tractography is based, and poor alignment with neighboring profiles. Recently developed contextual processing techniques, including enhancement and well-posed geometric sharpening, have shown to result in sharper and better aligned diffusion profiles. However, the research that has been conducted up to now is mainly of theoretical nature, and so far these techniques have only been evaluated by visual inspection of the diffusion profiles. In this work, the method is evaluated in a clinically relevant application: the reconstruction of the optic radiation for epilepsy surgery. For this evaluation we have developed a framework in which we incorporate a novel scoring procedure for individual pathways. We demonstrate that, using enhancement and sharpening, the extraction of an anatomically plausible reconstruction of the optic radiation from a large amount of probabilistic pathways is greatly improved in three healthy controls, where currently used methods fail to do so. Furthermore, challenging reconstructions of the optic radiation in three epilepsy surgery candidates with extensive brain lesions demonstrate that it is beneficial to integrate these methods in surgical planning.

## Introduction

With diffusion MRI (dMRI) and tractography the architectural configuration of white matter fiber bundles can be investigated in vivo, offering new avenues in neurosurgery, brain research, and the evaluation of brain disorders. dMRI tractography faces some outstanding challenges in such applications, though. Amongst these are improvements in the geometry of the diffusion profiles on which tractography is based, such as reduction of noise and alignment with neighboring profiles. Contextual processing techniques aim at enhancement of structures seen in diffusion MRI data, while reducing high frequency noise and small incoherent structures by using information from the local context. This can be used to enhance input for subsequent analysis such as fiber tracking.

As dMRI data contains both spatial (3D position coordinates) and angular (diffusion properties in different directions per position) information, contextual processing techniques can use either spatial, angular, or both spatial and angular information of the data. Spatial techniques, such as (anisotropic) smoothing, use the spatial context for a given direction and, hence, are applied directly to the individual DW images (DWIs). This has shown to be beneficial in detecting microstructural changes [1]. Angular techniques (e.g. [2]) use diffusion information in all directions at each individual position, for example to sharpen diffusion profiles [3]. Neither of these approaches includes the notion of *alignment* of neighboring diffusion profiles over the joint space of positions and orientations. To determine whether a diffusion profile is well aligned with a surrounding profile, one applies rigid body motions. The Lie group of rigid body motions (or 3D special Euclidean motion group, 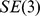) in fact naturally induces such a coupling between positions and rotations: 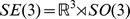, where 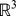 is the 3D position space and 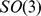 the 3D rotation group (see Appendix S1 for an overview of used symbols and abbreviations). Here, the semidirect product 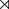 stresses the coupling. The coupled space of positions and orientations 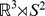 (with 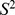 being the 2-sphere) is a so-called Lie group quotient embedded in 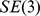 [4] (formally, this space is defined by 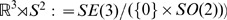). The type of processing that employs this coupling, acting on the joint coupled space of positions and orientations, will be called contextual processing on 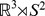.

Several techniques have been developed for contextual processing on 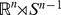 (with n denoting the dimension), including [5]–[10] for 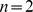 and [4], [11]–[18] for 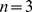. The works of [4] and [19] describe the general case of enhancements (i.e., convection-diffusion operators) and erosions, which are respectively forward Kolmogorov and Bellman equations for Brownian motions on 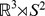. These works include special cases considered in interesting other works [11].

The enhancement methods described in [4] have shown to be able to extrapolate reasonable fiber crossings from diffusion tensor imaging (DTI) data, and to remove spurious crossings in high angular resolution diffusion imaging (HARDI) data [14], [20]. In their work, all legal linear convection-diffusion operators on 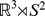 are expressed in a moving frame of reference. The methods were implemented using (left-invariant) finite differences [13] or convolution with a kernel corresponding to the convection-diffusion operator [20]. These convolutions further generalize other contextual processing techniques such as tensor voting and channel smoothing, in the sense that they allow multiple orientations per position.

On top of the convection-diffusion operations, erosions on 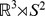 have recently been developed to sharpen the diffusion profiles [15], [19], serving as a preprocessing step for convection-diffusion processes. The work that has been done up to now is mainly theoretical in nature, and visual inspection of the diffusion profiles was performed on a real human brain data set only. To demonstrate the usefulness of these methods in (clinical) applications, further qualitative and quantitative evaluation is needed.

In this work, contextual processing methods on 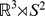 are evaluated in a clinically relevant application: The reconstruction of the optic radiation (OR) for epilepsy surgery. Epilepsy surgery is considered when patients still have seizures that influence their life intensely despite medication. A common complication is a visual field deficit [21]–[23] due to disruption of the OR. In the case of severe visual loss, the patient is not allowed to drive anymore, which reduces the quality of life in these patients to a great extent [24]. Since there exists a large intersubject variability in the location of the OR and especially its anterior extent (called the Meyer's loop) [25], assessing the risk of visual loss is complicated and therefore localizing and visualizing the OR is useful [26].

**Figure 1:**
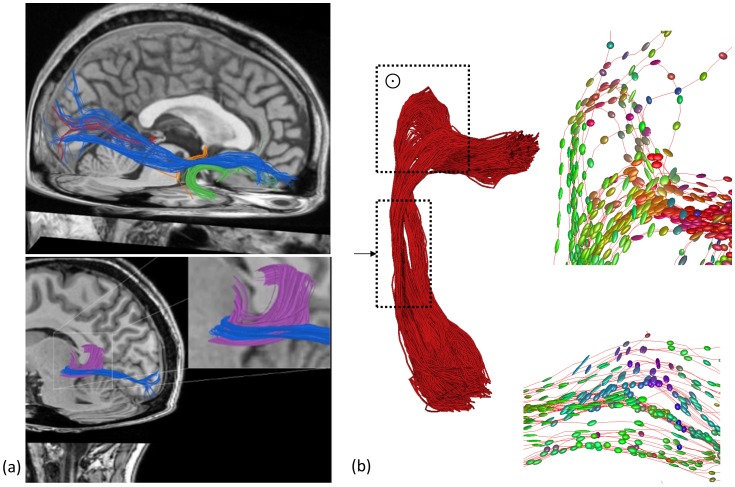
Optic radiation tractography (a). Top: Spatial relationship between the OR (red), uncinate fasciculus (green), anterior commissure (orange) and inferior longitudinal fasciculus (blue). Bottom: Position of the tapetum of the corpus callosum (purple) can be seen in relation to the OR (blue). (b) Regions of complex fiber architecture of the OR. Right top: Diffusion tensors around areas of high curvature in the Meyer' s loop. Right bottom: Diffusion tensors in the top middle point in inferior-superior direction, indicating crossing fibers.

Several studies used DTI to reconstruct the OR pathways [26]–[38]. Reconstruction of this particular white matter fiber pathway is complex, however, since it is highly curved and closely located to other fiber pathways (Fig. 1). In particular, reconstructing the curved Meyer's loop appears to be extremely challenging. Deterministic tractography often leads to an underestimation of the anterior extent of Meyer's loop compared to dissection studies [28], [29], [35]. This disability is attributed to the presence of crossing fibers near the most anterior part [29] or difficulties tracking fibres thinner than the voxel size at the edge of Meyer's loop [28]. Probabilistic tractography, compared to deterministic tractography, attempts to cope with data uncertainty by defining a distribution of fiber directions at each position [39], [40] and can better delineate Meyer's loop [27], [35], [36], [41]. These methods generate a large amount of pathways and provide more insight into uncertainty of the propagation of tracts. The question remains, however, how reliable each individual pathway is. Sherbondy et al. (2008) [41] separated the sampling of pathways between two points that are assumed to be connected from assigning a ‘plausibility’ assessment (i.e., score) to them, in order to consider the validity of each possible pathway independently. In this way, their ConTrack algorithm could reconstruct pathways that were previously missed, and they were able to reconstruct the anterior extent of the OR (Meyer's loop).

For evaluation of the contextual processing techniques on 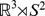 in OR reconstruction, we have developed a framework in which we incorporate a scoring procedure for individual pathways. The ConTrack scoring measure, however, is limited to DTI data and involves many parameters that are generally hard to interpret geometrically. Moreover, these parameters have a complex relationship with the pathway estimates. Therefore, we have developed a new scoring measure based on a sub-Riemannian distance on 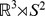. The scoring measure consists of a data dependent part, and a data independent part that takes into account geometrical properties of the pathway. The evaluation framework includes the following steps: after tracking a large amount of pathways with a probabilistic algorithm on the initial (non-contextually processed) tensor data, the data is ‘transformed’ to 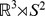, and erosions and diffusions are applied to the data [19]. Subsequently, each individual pathway is assigned a score based on this processed data. We evaluate the pathways by setting a threshold on the score and look at the highest scoring fibers, which ideally represent reliable and anatomically plausible fibers. We will show that our scoring measure can discriminate best between anatomically plausible and implausible fibers when both erosions and diffusions are applied, compared to no contextual processing. We qualitatively and quantitatively evaluate our framework on the reconstruction of the OR in three healthy controls. In addition, a comparison is made to the ConTrack scoring [41].

In this study, the framework was also applied for patients for whom epilepsy surgery was considered because they had seizures that influenced their life intensely despite medication, while the epileptogenic region was presumably adjacent or overlapping with the cortical visual areas. The application of the framework in these epilepsy patients showed that even in case of highly disrupted anatomy due to these lesions we could extract plausible fiber pathways that most likely represent the (remainder of the) OR. These results may play a role in the guidance of surgical planning in these patients. Localization of the seedpoints for tracking was guided by functional MRI (fMRI) activity maps obtained by visual stimulation to increase the accuracy and reproducibility of the tracts [42], [43].

This paper is organized as follows. First, a brief background on contextual processing on 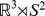 is given, and we will explain the so-called *coupling* of positions and orientations in the Theory section. In the Materials and Methods section, the evaluation framework and scoring measure are presented.

## Theory

**Figure 2:**
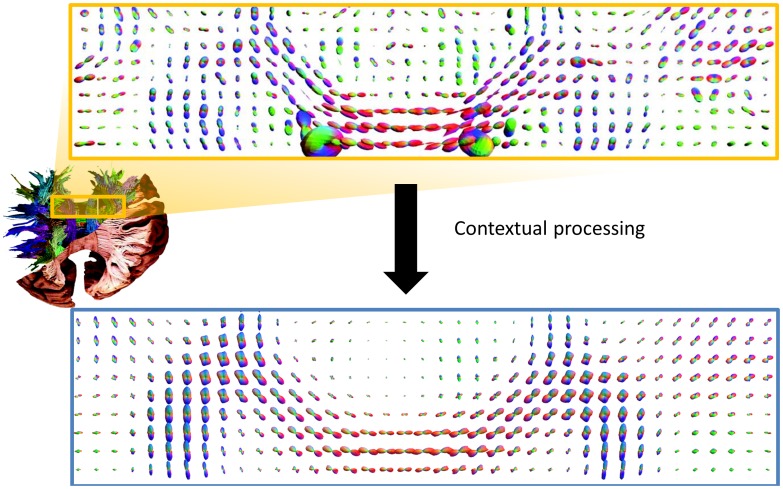
Result after contextual processing on 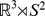 on DTI data in a coronal slice. Diffusion profiles are better aligned, sharper, and crossing fiber regions can be recognized.

dMRI data suffers from a high noise level which affects any model or quantity that is estimated from the data. Noise reduction by smoothing can be pursued to increase the accuracy of dMRI processing. While suppressing noise, it is important to preserve or ideally enhance important structures present in the data. Therefore, enhancement techniques have been developed which are promising in clinical applications [44]. Difficulties still remain, as enhancement lowers the level of detail. To remedy this, one can use sharpening methods that emphasize prominent peak directions. Contextual processing techniques that consider spatial and directional context together instead of either spatial or directional context alone, have shown to perform particularly well at locations where fibers cross or bifurcate [4], [12]–[14], [19]. In our framework, dMRI data and 3D curves (i.e., fiber pathways) are considered to live in the *coupled* space of positions (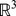) and orientations (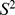). Fig. 2 shows the performance of these methods in an example slice, where noise is reduced and diffusion profiles are better aligned. We will first provide some background on the coupled space, and subsequently explain the enhancement and sharpening methods used in our framework.

### The coupled space of positions and orientations

#### Oriented random walkers and their evolution

**Figure 3:**
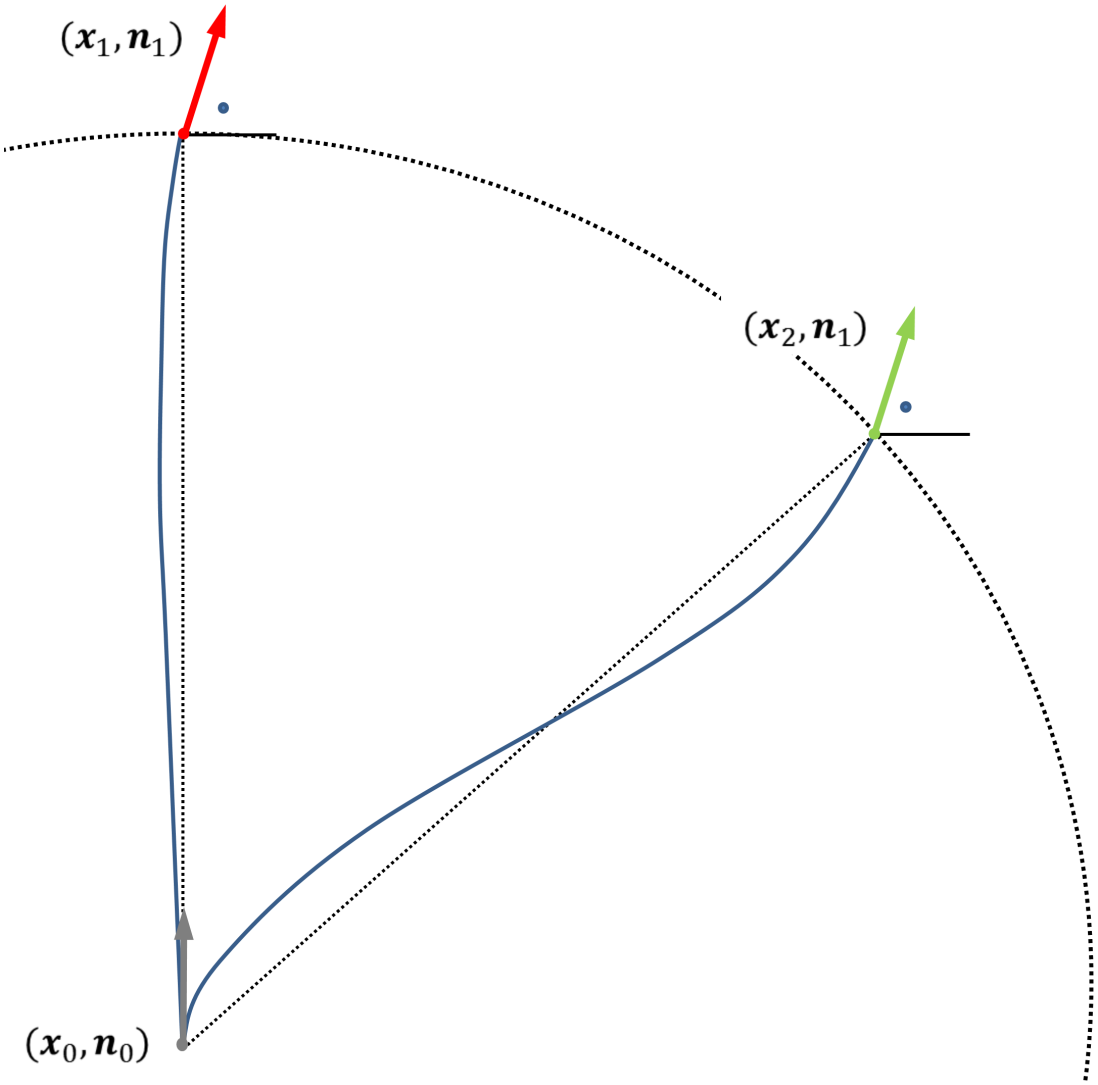
Coupling of position and orientation. Particle 0 has position and orientation 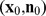, particle 1 and 2 are denoted by 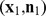 and 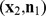 respectively, thus having a the same spatial distance from particle 0 (denoted by the dashed circle) and the same orientation (indicated by the dots). The solid lines represent the sub-Riemannian distance (here the unique global minimizing geodesic in 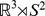 connecting two oriented particles) from particle 0 to particle 1 and 2 respectively, where the former is shorter.

Consider an ‘oriented’ particle located in 3D space 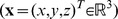 that has an orientation 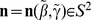, with 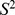 the 2-sphere. Here, we parameterize the sphere by angles 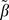 and 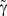 so that each orientation can be parameterized as
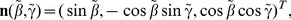
(1)with 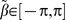 and 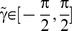. In contextual processing, it is important to check for ‘aligned’ particles in the surroundings. To define the degree of alignment between particles, the metric that defines ‘distance’ (sub-Riemannian distance, see [6], [19], [45]–[47] for the 2D case and [48] for nD) in this space should incorporate both spatial and directional ‘distance information’. Consider Fig. 3: Suppose particle 1 at 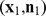 and particle 2 at 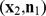 have the same orientation and we want to check for alignment with particle 0 at 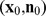 that is on an equal spatial distance 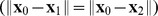 from both particles. When no *coupling* would be implied between the spaces 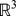 and 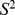 and we would have considered 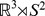 as a flat Cartesian space, there would be no difference between the sub-Riemannian distance of particles 1 and 2 with respect to particle 0. After all, they have the same spatial and angular distance (angle) to particle 0. When considering spatial and angular information together (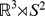, we write 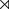 here to stress the coupling), one can see that the sub-Riemannian distance between particle 1 and 0 is shorter (here represented by the length of the line connecting the two oriented particles, which is a geodesic in 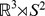). From this it can be concluded that treating 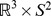 as a Euclidean space with Euclidean norm does not allow to distinguish between well aligned and poorly aligned oriented particles, whereas 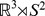 does allow such a distinction (see also [19]).

The evolution of this oriented particle or random walker in the coupled space of positions and orientations can be described by stochastic differential equations [19], [49]. To ensure rotation and translation covariance, it is convenient to express the partial derivatives in these differential equations in a local frame of reference attached to an oriented particle, in which we can describe translations and rotations with respect to that particle. Take for example particle 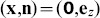 with 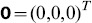 and 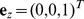, where a local frame of reference is given by
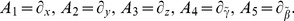
(2)


**Figure 4:**
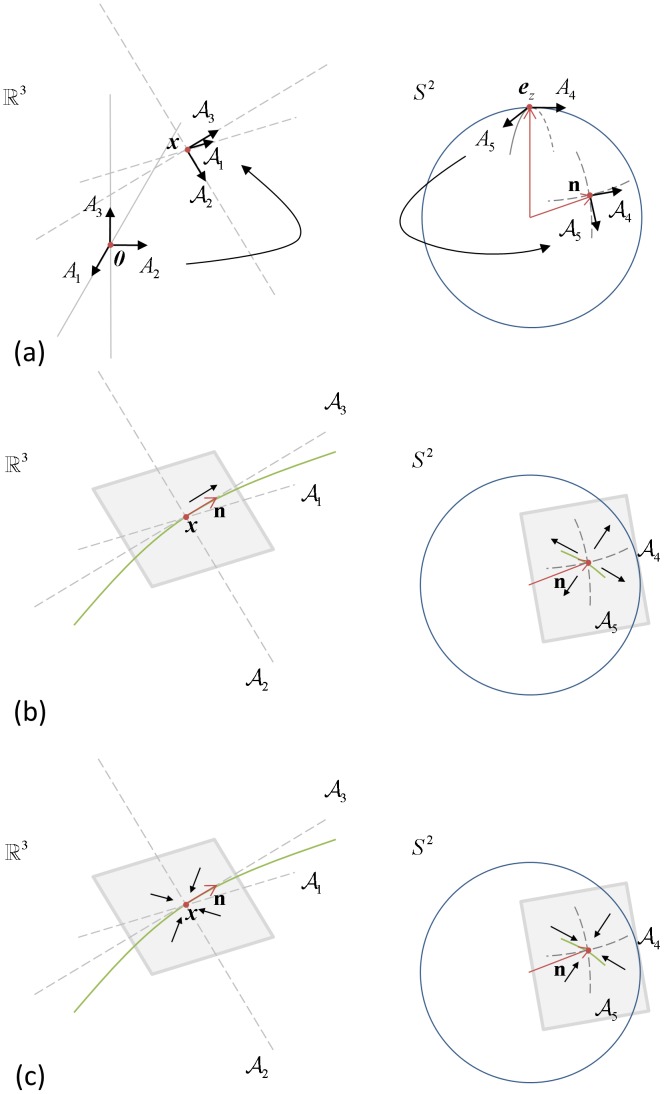
Local frame of reference, diffusions, and erosions on 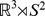. (a) Local frame of reference for 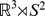. 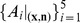 is the frame for particle 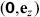, and 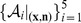 is the frame attached to a general particle 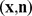. (b) Spatial diffusion takes place along 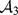 in space and angular diffusion (outward) in the tangent plane spanned by 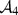 and 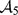. (c) Geometrical erosion takes place both inward in the tangent plane spanned by 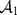 and 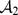 in space and inward in the local tangent plane spanned by 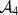 and 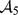.

This is visualized in Fig. 4(a) for the spatial (left) and directional (right) partial derivatives, note that 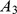 is in the spatial direction of the random walker. A local coordinate frame at every other oriented particle 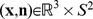 can then be obtained by translation and rotation 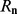 of 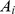:
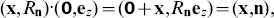
(3)and is denoted by 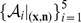, so 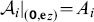. We choose 
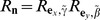
, with 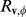 denoting a counter-clockwise rotation 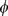 about rotation axis 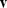 (note that 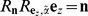, but since we consider legal operators the choice of 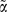 is not crucial in our algorithms as explained in [19]). Analytical expressions for these so-called left-invariant derivatives in charts of Euler angles can be found in [4] and without Euler angles in Appendix S2.

#### Distribution of oriented random walkers and its evolution

Now suppose we have a distribution of oriented particles 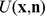 that gives the probability density that a particle starts at position 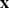 *and* travels in direction 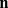. We will see that the diffusion profiles can be regarded as probability density 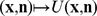, and that enhancement and sharpening processes can be written as evolution equations on probability densities 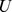. This probability density can directly be associated to the stochastic process 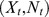 on 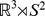 describing the movement of water molecules in brain white matter during time 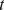. Here the random variable 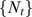 denotes the direction of a random walker in 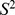, and 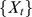 its position in 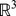 at time 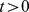. According to the conditional probability function, we define
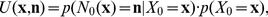
(4)where 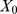 is the starting position of the particle. The term 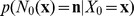 in Equation (4) is generally called the orientation density function (ODF), which can be obtained by radially integrating the diffusion probability density function (PDF) 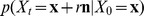 at each location, where 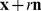 is the ending position of the particle, 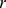 the distance to the start point, and 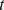 the diffusion time. The factor 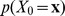 in Eq. (4) represents the probability density of finding an oriented particle at 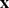 at time 0, regardless of its orientation.

Evolution of distributions 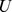 of oriented particles can also be described by partial differential equations (PDEs). Our framework includes enhancement and sharpening steps, and we will discuss the diffusion equation for linear contour enhancement and the erosion equation for well-posed sharpening of the diffusion profiles in the next sections.

### Diffusion equation for linear contour enhancement

To enhance aligned elongated structures (corresponding to fibers) in the data, we apply diffusion on 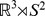. The diffusion equation for the stochastic process of countour enhancement that will be considered is a Fokker-Planck equation on 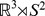 [4], [19]:
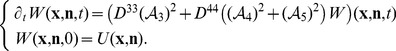
(5)


Here 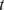 is the stopping time, 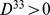 determines the diffusion along 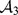, and 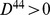 the isotropic angular diffusion. Both parameters also have a probabilistic interpretation: they equal half the standard deviation of a random walk in the 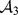 and isotropic angular directions, respectively (see also [4], paragraph 10.2). The underlying geometrical idea of contour enhancement is visualized in Fig. 4(b). The depicted curve represents any potential curve passing through 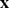 with orientation 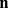. Spatial diffusion takes place along 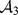 in space and isotropic angular diffusion (outward) in the tangent plane spanned by 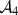 and 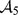.

### Erosion equation for sharpening

The diffusion process not only aligns but also broadens the distributions, so some spatial and angular sharpening process is needed. Sharpening of the diffusion profiles can be obtained by nonlinear greyscale transformations such as squaring [14], [20]. However, global maxima are very dominant and large isotropic diffusion profiles will be amplified. Erosions provide a way to sharpen the diffusion profiles in a more controlled way, by setting the amount of spatial and angular sharpening. The partial differential equation for erosion, with parameters 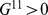 and 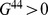, is a Hamilton Jacobi Bellman equation for a cost process on 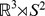, given by (see [19], paragraph 7.2, theorem 8 and 10):
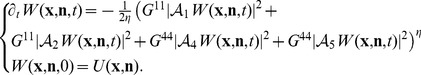
(6)


Where the fraction 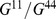 determines the amount of spatial erosion relative to angular erosion, and 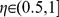 determines the homogeneity of the Hamiltonian (and Lagrangian) [15], [19]. Angular erosion (controlled by 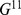) considers sharpening of orientation distributions per position, whereas spatial erosion (controlled by 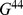) considers sharpening of spatial distributions per orientation. Fig. 4(c) illustrates that such geometrical erosion takes place both inward in the spatial tangent plane spanned by 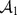 and 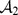 in space and inward in the local angular tangent plane spanned by 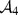 and 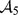.

## Materials and Methods

**Figure 5:**
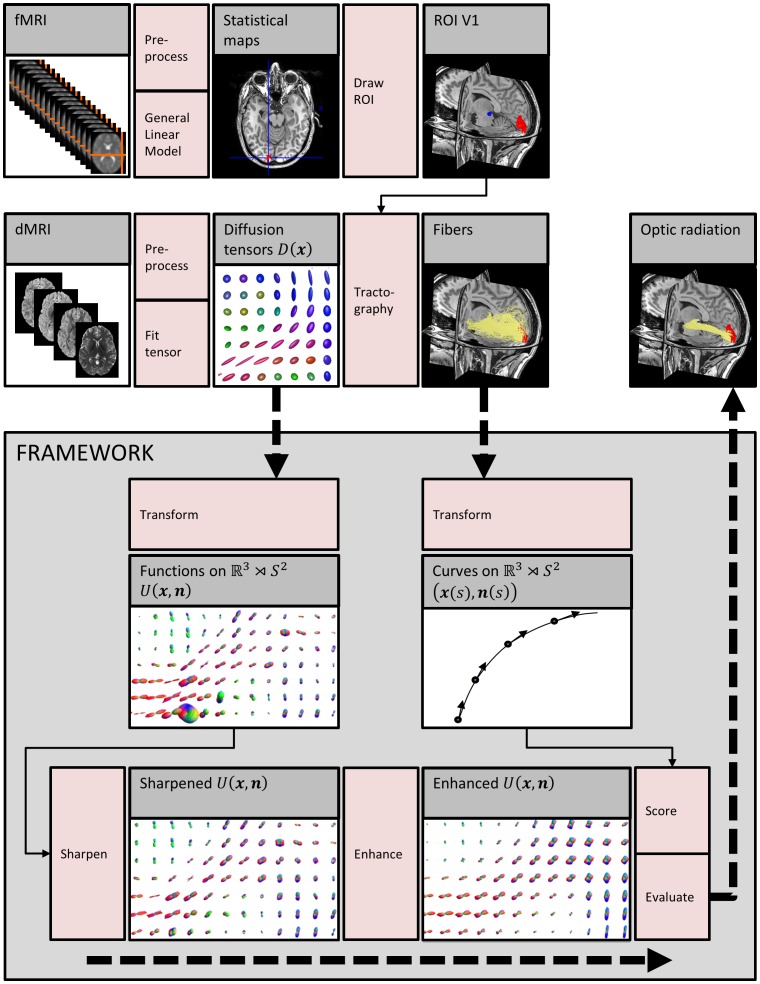
Evaluation pipeline. The top row represents the fMRI pipeline to extract a ROI for seeding of the tractography in V1, whereas the middle row displays the dMRI pipeline. The tensors and fibers are input to the framework that contains the contextual processing on 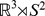 (sharpening and enhancement), and subsequent scoring and evaluation.

In this section, we will present our framework for evaluation of contextual processing on 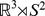. An overview of the whole pipeline can be found in Fig. 5. In addition to dMRI data, we acquired fMRI data to localize the primary visual cortex both in healthy controls and patients. After specifying experimental settings, we will outline briefly how we localized the region of interest (ROI) in V1 based on the fMRI activity maps and the ROI in the lateral geniculate nucleus (LGN) based on the anatomical scan. Subsequently, we discuss the dMRI pipeline represented by the second row of Fig. 5, after which we end up with a large amount of fibers resulting from probabilistic tracking. Finally, the contextual processing evaluation framework is explained in detail, displayed schematically in the grey box in Fig. 5. The framework has as input the diffusion tensors and the large amount of probabilistic fiber pathways, and outputs the highest scoring fibers.

### Subjects

The study had the approval of the Medical Ethics Committee of Kempenhaeghe and all participants gave written informed consent for the study. The data of three healthy volunteers (female, age 22, 22 and 47) was used to optimize the directional contextual processing and scoring procedure for reconstruction of the OR. Subsequently, we applied the framework to three patients who were candidates for epilepsy surgery. Extensive pre-surgical assessment of the epilepsy of these patients was performed at Epilepsy Centre Kempenhaeghe, including a structural 3T MRI confirming anatomical abnormalities. All patients underwent extensive standard electroclinical studies, including a long term presurgical video-EEG seizure monitoring session. As a result of these studies it was assumed that the epileptogenic zone was related to the lesion visible on MRI, leading to the clinical hypothesis of the epileptogenic lesion being the cause of the epilepsy of the patient. Patient 1 is a 25-year old man who has had a infarction of the medial cerebral artery after birth resulting in a cystic encephalomalacia in the right hemisphere. Static automated perimetry using a Humprey field analyzer demonstrated that the patient had a lower quandrantanopsia. Patient 2 is a 34-year old woman who had a perinatal bleeding resulting in an extensive cystic lesion in the left hemisphere. According to perimetry results this patient was not suffering from visual loss. The MRI of patient 3 (age 42) revealed a cavernoma in the occipital region of the left hemisphere. According to perimetry results, this patient was not suffering from visual loss either. All three patients were candidates for epilepsy surgery, patient 1 and 2 for a functional hemispherectomy, while patient 3 was candidate for resective surgery. As part of the epilepsy surgery decision making process for all three patients included it was questioned whether vision was affected by the epileptogenic lesion and whether the planned resection of the lesion was going to affect vision. These patients, therefore, were referred for fMRI to test their visual function. Additionally, the experimental dMRI study was performed to reconstruct the OR and predict possible damage to the OR as result of the planned resection. Although the question for all three patients was whether surgery would lead to additional visual loss, the patients were notified that this study was not part of the standard pre-surgical evaluation and that it did not influence any of the clinical decisions, such as the surgical resection.

### Data acquisition

All scans were acquired on a 3.0 T Philips Achieva MR scanner equipped with a SENSE-head-8 receiver coil. The scanning protocol consisted of an anatomical T1 image, an fMRI scan to locate V1 for tractography seeding, and one DTI scan.

For the fMRI image, a GE-EPI (Gradient-Echo Echo-Planar Imaging) was used for rapid acquisition of the whole brain. Timing parameters were TE  = 35 ms and TR  = 2500 ms. 24 slices were scanned per volume with thickness 4 mm and an acquisition matrix of 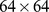 with in-plane pixel size of 4 mm leading to a field of view (FOV) of 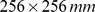.

The DWIs were scanned using a Single-Shot Spin-Echo Echo-Planar Imaging (SE-EPI) sequence, scanning 59 axial slices with a slice thickness of 2 mm. An 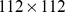 acquisition matrix was used that covered a FOV of 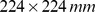 leading to isotropic voxels of 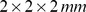. SENSE  = 2, leading to a reduction of the effective FOV by a factor 2. Diffusion sensitizing gradients were applied in 32 directions uniformly distributed over the sphere [50], [51] with a b-value of 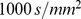 along with one b = 0 image. The scan took around 8 minutes, using TE/TR  = 73/6718 ms and number of signal averages (NSA)  = 2. Data of patient 1 and 2 were available with a slightly different protocol with the same b-value and resolution, using TE/TR  = 55/10254 ms and NSA  = 1. The same amount of gradient directions was used with the directions distributed according to the standard clinical protocol of the Philips scanner.

### Seedpoint selection for fiber tracking

#### fMRI pipeline: localization of V1

**Figure 6:**
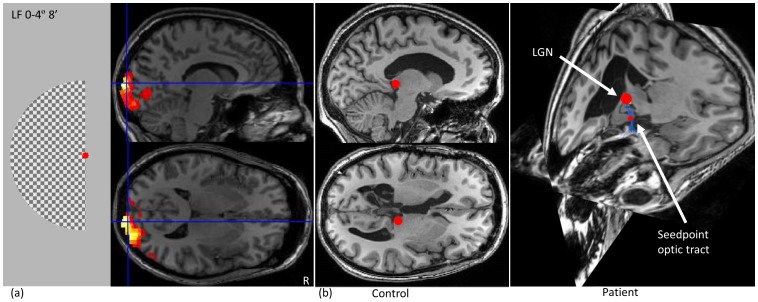
Seedpoint selection for tractography. (a) fMRI activity maps in V1 resulting from visual stimulation with a left half field stimulus (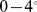 denotes the eccentricity angle, and 8′ denotes the check size in arc minutes. The size of the red fixation point is 10′). Drawing the seedpoint in V1 was guided by this activity map. (b) Localization of the LGN in healthy controls (left) and patients with disrupted anatomy due to a lesion (right). In patients, the LGN was localized by tracking the optic tract via a manually drawn seedpoint.

There is a large variation in the exact location of V1 in healthy subjects [52], and it is not straightforward to localize V1 in patients with extensive brain lesions or earlier resections. The seedpoint in the primary visual cortex was therefore based on the Blood-Oxygen-Level-Dependent (BOLD) fMRI activity maps resulting upon visual stimulation (Fig. 6(a)) with a half-field square checkerboard pattern-onset stimulus that selectively activates V1 [53]. The subjects were asked to focus on the red fixation point both during activation and rest. An epoch related design was used with an activation period of 20 s and a resting period of 20–25 s, and the paradigm consisted of ten activation and resting periods. The individual scans were realigned, registered to the anatomical T1 (rigid), and smoothed with a Gaussian kernel with full-width-half maximum of 8 mm using SPM5 [54]. Alignment of the scans was confirmed upon careful visual inspection. A general linear model was used to localize areas of significant activation. From these maps, a connected region was manually drawn that served as seedpoint for fiber tracking.

#### Localization of the LGN

The LGN was manually located on the anatomical T1 for the healthy controls as can be seen on Fig. 6(b) left, using a sphere of 4 mm radius (volume of 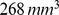, the LGN has a volume of 91 to 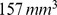 [33]). In the patients with severe malformations, the LGN was very hard to locate on the anatomical T1 image. A seedpoint sphere of radius 2 mm was placed in the optic tract (Fig. 6(b) right), which was clearly distinguishable at T1. A basic deterministic (streamlines tracing technique Runge-Kutta 4) fiber tracking algorithm implemented in mrDiffusion (VISTA lab, Stanford University) was used to track fibers from this seed region [33]. The step size was 1 mm, angle threshold 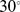, and FA threshold 0.2. The termination point of the fibers was assumed to be the location of the LGN.

### dMRI pipeline

#### Preprocessing and tensor fitting

The preprocessing of the DW MRI data prior to the fitting of a model was performed with mrVista (VISTA lab, Stanford University). This included motion- and eddy current distortion correction [55], rigid registration to the T1 image aligned to AC-PC space [56], and proper rotation of the B-matrix [57]. The diffusion tensors were estimated using a linear least squares approach. A residual bootstrap procedure [33], [58] generated 1000 permutations of the raw DW data and resulted in a principal diffusion direction dispersion map to account for uncertainty in the data, which was used in the probabilistic fiber tracking. Finally, a white matter mask was generated by requiring either 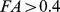 regardless of mean diffusivity, or 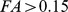 together with a mean diffusivity less than 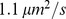, and subsequent dilation of one voxel [41].

#### Tractography

For fiber tractography, the sampling step of ConTrack was used [41]. From a seedpoint that is randomly placed in one of the two ROIs, the pathway is grown in iteratively chosen step directions: When the fiber direction uncertainty is small (as derived from the bootstrapping procedure), the next direction is a random sample form a Bingham distribution that reflects how well a given direction is supported by the underlying diffusion data. Otherwise, the next direction is sampled from a distribution that incorporates the length and smoothness of the curve (based on a Watson distribution). The algorithm tracked 100 000 tracts between the two seedpoints (50 000 in patients 1 and 2 because of their extensive cystic lesions).

### Contextual processing and scoring

We used two approaches for the sharpening and subsequent enhancement: one that is in line with the work of [14] and [4] (approach A), and one that explores the methods used in more recent work of [19] (approach B). This is summarized in Table 1. Each processing block of the framework in Fig. 5 is further explained in the following sections. The contextual processing framework was implemented in Mathematica 8 (Wolfram) and can be downloaded from http://bmia.bmt.tue.nl/people/RDuits/. For visualization of the results, VIST/e (http://bmia.bmt.tue.nl/Software/vISTe/) was used.

**Table 1:** Overview of the approaches for contextual processing on 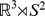.

Approach		Normalization	Sharpening method	Enhancement method	Normalization
Initial	No sharpening and enhancement				
A	Sharpening only		square		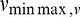
	Sharpening and enhancement		square	convolution	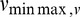
B	Sharpening only	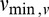 and 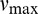	erosion		
	Sharpening and enhancement	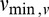 and 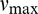	erosion	finite differences	

In the ‘initial’ condition, fiber pathways are scored on data without contextual processing. Approach A uses sharpening by squaring and subsequent enhancement by convolution with the kernel. Approach B uses erosions for sharpening and finite difference schemes for enhancement. Normalizations 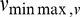 and 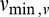 indicate min-max and min normalization per voxel respectively, whereas 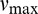 scales diffusion profiles by the global maximum.

#### Transformation of diffusion tensors to a function on 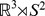


For the transformation of DTI data to functions 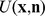, Eq. (4) is used [13], [19]. The term 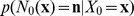 is the ODF and is obtained by radial integration of PDF which is modeled as a 3D Gaussian distribution in DTI,
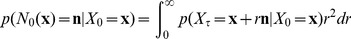
(7)

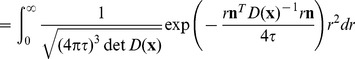


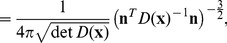
(8)for each position 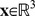, with 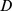 the diffusion tensor field. For the spatial probability 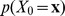 we propose in analogy to [13]:
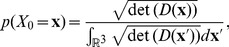
(9)where 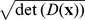 is proportional to the volume of the ellipsoid representing the diffusion tensor 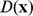, which is 
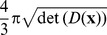
. Note that the term in Eq. (9) cancels the 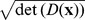 term in the nominator of the ODF expression.

Diffusion tensors were converted to 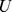 using a icosahedron of order 2 as tessellation. This results in a discrete spherical function with 92 directions.

#### Contextual processing

In this section, we will specify the implementation and parameters used for the different contextual processing approaches, summarized in Table 1. To be able to compare the intermediate sharpening steps with the results after subsequent enhancement, the data were normalized in the same way before evaluation.

**Approach A** is analogue to the work of e.g. [4]. Sharpening is obtained by squaring the input data 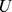 [14]. For linear contour enhancement an approximation of the Green's function of the diffusion equation was used, see Appendix S2. This kernel 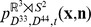 was precalculated using parameters 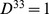, 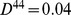, and 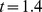 [20]. The enhancement was performed via 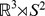 convolution (Appendix S2). The result was min-max normalized before scoring.

**Approach B** uses the methods developed in [19]. Here, the data was sharpened using erosions with parameters 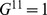, 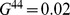, 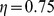, 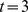 and step size 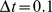, after subtracting the minimum per diffusion profile (locally) and dividing by the global maximum of the data [15], [19]. Erosions are implemented using upwind schemes [15], [19]. Linear contour enhancements are done by approximating the diffusion equation by standard centered second order finite differences and using a forward Euler scheme for time discretization [13], see Appendix S2 for a summary. Parameters were 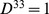, 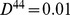, 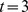 and step size 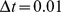. For the angular and spatial step sizes 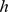 and 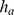 we used 0.88 and 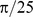, respectively.

#### Transformation of curves to 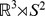


To transform the reconstructed pathway tracts to curves in 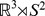 so that they are in the same space as the diffusion data, we parameterize both the spatial and angular part of the curve 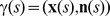 by the arclength parameter 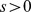 [59]. The orientation 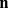 of a curve at a particular position 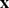 in arbitrary parameterization 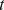 is the orientation of the unit tangent, i.e.
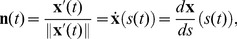
(10)which can be calculated analytically in each point from the polygon obtained from cubic spline interpolation of the curve [60].

#### Scoring pathways

The scoring measure should both encapsulate how well a pathway is supported by the data and also incorporate knowledge about the geometry of the pathway tract. We propose the following scoring measure, which can be derived from Bayesian inference (Appendix S3):
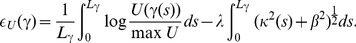
(11)


**Figure 7:**
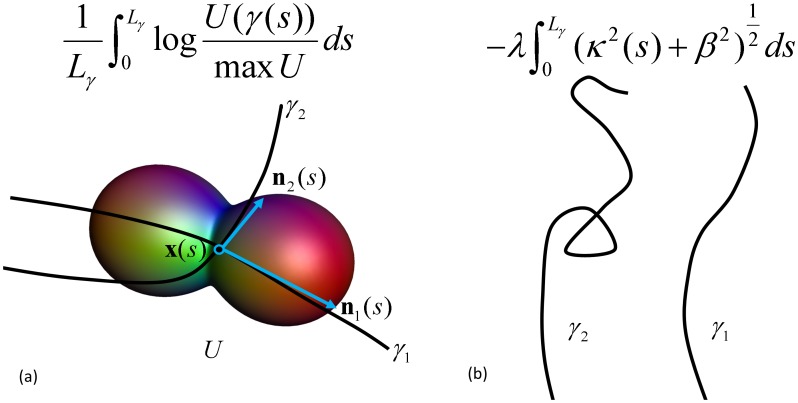
Illustration of the scoring term, with 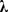 the weight between internal and external energy. (a) External energy or data dependent term, 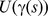 represents the function 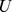 at a particular position 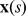 evaluated in the direction of the tangent vector of the fiber 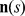. In this example, 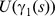 has a larger value than 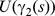. (b) Internal energy or data independent term, punishes length (by the parameter 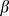) and curvature 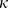 so that 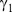 is preferred over 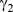.

The first term on the right hand side represents the external energy or data dependent part which takes into account the underlying diffusion information. It reflects the nearest neighboring function on positions and orientations evaluated in the direction of the pathway as illustrated in Fig. 7(a). This value is taken relative to the maximum value in the data set. The multiplication by 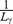 avoids preferring short tracts to long ones, where 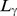 is the length of the curve. Furthermore it naturally averages the contribution of the data dependent term in Eq. (11). Trilinear interpolation is used to evaluate 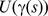.

In the second term, which represents the internal energy or data independent part, it is possible to incorporate previous knowledge about pathway geometry. Here, 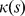 is the curvature of the curve and 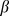 is a parameter that can be tuned to penalize or reward length (typical value between 0.01 and 0.1), see Fig. 7(b). Parameter 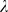 defines the ‘weight’ of the internal energy with respect to the external energy, and can be set to zero when one only wants to evaluate the fit to the data. Curvature in each point was calculated via the Frenet-Serret formulas to each polygon resulting from cubic spline interpolation of the pathways [60]. In most cases, scoring based on the data dependent term was sufficient to obtain plausible reconstructions (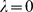), unless indicated otherwise.

In the evaluation of the integral, the step size 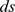 is approximated by 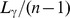, where 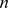 is the number of sample points of a pathway. The first and last point of a pathway were not taken into account, since it is expected that these are often located in the grey matter. For the healthy controls, a random subset of 10 000 fibers was used for the experiments for the evaluation of both contextual processing approaches (A and B), and for the rest of the results all pathways are used.

Our framework is compared to the ConTrack scoring step [41]. This scoring measure has a data dependent and data independent part, and uses the same distributions as the sampling step. The data dependent term uses a Bingham distribution with the dispersion parameters depending on the data uncertainty as a result of the bootstrapping procedure and the eigenvalues of the diffusion tensor. Parameter 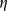 determines the effect of the linearity index 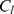 on the score. The data independent part incorporates the length of the pathway, where 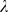 is a parameter to punish length and 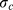 is related to dispersion parameter of the Watson distribution that punishes curvature. For details of the scoring, see [41]. We used ConTrack parameters 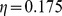, 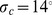 and 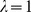.

#### Qualitative and quantitative evaluation of pathways

When we have assigned a score to each individual pathway using Eq. (11), we infer that the pathways with the highest score are the most likely. At a particular threshold, anatomically plausible fibers should be separated from anatomically not plausible pathways.

**Figure 8:**
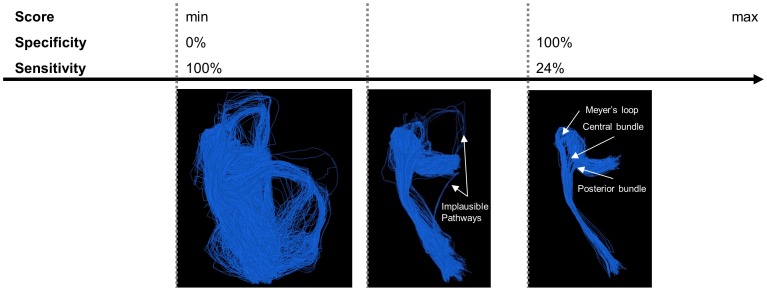
Illustration of the qualitative evaluation of the OR reconstruction, the selection of anatomically plausible fibers. When increasing the threshold score, more and more fiber pathways will be removed. At a certain point, all false positives will be removed and the specificity will be 100%. The sensitivity will be expressed as the percentage of remaining pathways, and should be maximized. In the reconstruction on the right, all visible pathways are anatomically plausible at specificity of 100%.

Qualitatively, the anatomical plausibility of a pathway was evaluated by visual inspection, confirmed by an expert. Anatomically plausible pathways are pathways that fulfill anatomical constraints, i.e. pathways that are limited to the hemisphere of interest and curling around the temporal horn (Fig. 8). Anatomically plausible pathways that have a score higher than a chosen threshold are defined to be true positives. A false positive pathway is a pathway with a high score that is anatomically not plausible. We want to set a ‘critical threshold’ for which zero false positives remain (i.e., we impose a specificity of 1), while maximizing the sensitivity. Sensitivity is high when the number of true positives is large and not too many anatomically valid pathways are thrown away (small number of false negatives). To put high standards, the method is considered to be inadequate in the case that for a specificity of 1, sensitivity is 0.

Quantitatively, we evaluated our reconstruction by comparing the Meyer's loop to the temporal pole (ML-TP) distance and Meyer's loop to inferior horn (ML-IH) distance to the ranges given in [25], where they reported 22 to 37 mm and −5 to 10 mm for ML-TP and ML-IH respectively. For the latter, a minus sign indicates that the OR lies more posteriorly.

## Results

In this section, we will show results of contextual processing (both approaches), and the scoring and evaluation step for one representative healthy control. The results for all three healthy controls are then given, where we compare our method to the ConTrack method. Finally, results of OR reconstructions for three patients with an epileptogenic lesion are presented.

### Healthy controls

**Figure 9:**
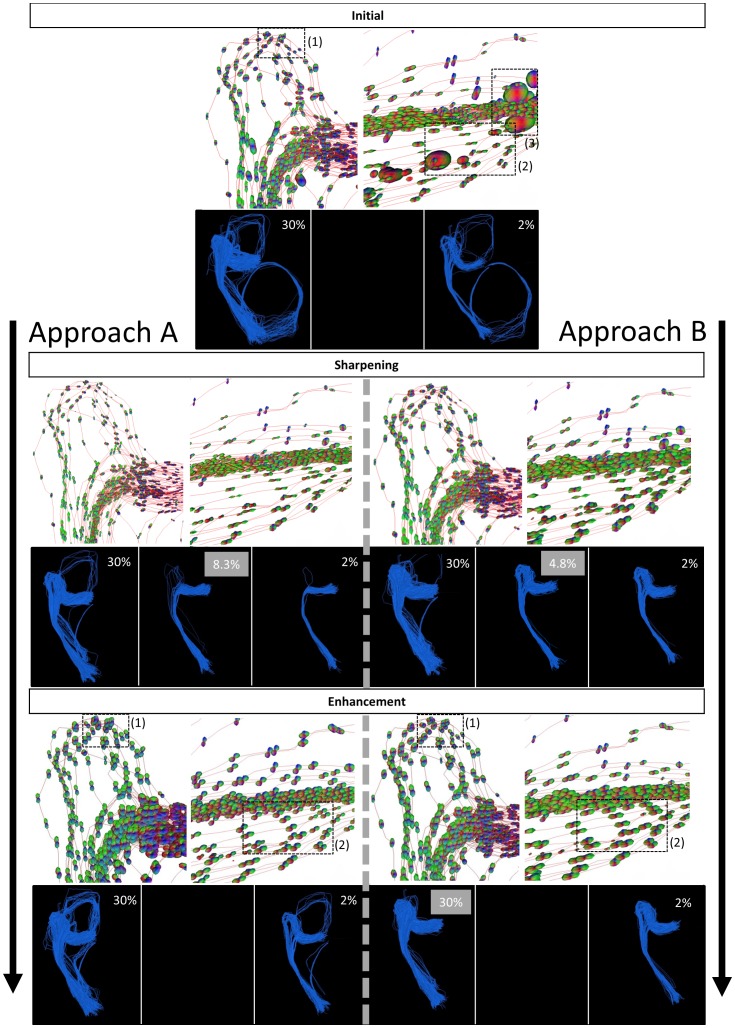
Results for scoring and qualitative evaluation of the OR reconstruction. Diffusion profiles are visualized along tracts that can anatomically be identified as OR pathways, randomly picked from the large set. We show 2%, 30% and (if existing) the percentage highest scoring fibers without false positives, highlighted in gray. Top: scoring directly on the original data, diffusion profiles are non-aligned with tracts of the OR (as indicated in region (1) and (2)). Partial volume effects with CSF cause the large diffusion profiles in (3), a white matter mask is applied in subsequent steps. Approach A (left): sharpening of the diffusion profiles by a gray-value transform (squaring) without enhancement (middle) and after enhancement (bottom). Approach B (right): sharpening of the diffusion profiles by erosion without enhancement (middle) and after enhancement (bottom). Note that diffusion profiles are globally scaled for visualization purposes.

Regarding the qualitative evaluation, Fig. 9 shows the results of contextual processing and subsequent scoring in a flowchart, corresponding to Table 1. Every row shows intermediate results, and for every result the diffusion profiles around the Meyer's loop and the straight part of the OR are visualized (corresponding to Fig. 1(b)), as well as the result of scoring and evaluation. For the latter, we always show the 30% and 2% highest scoring fibers, and the percentage at which no false positives remain (if it exists, it is highlighted in gray for clarity). We start off with the initial condition at the top of Fig. 9, where the diffusion tensors are transformed to functions on positions and orientations without further processing. Applying the scoring measure directly on this data results in many false negatives and it clearly leaves false positives when the score threshold is increased, meaning a critical threshold does not exist. This can be explained by the poorly aligned diffusion profiles around the Meyer's loop and other locations of the OR, which lowers the score of anatomically plausible OR pathways. For subsequent contextual processing, we split up in two approaches, approach A on the left and B on the right. For both approaches, the diffusion profiles are generally sharper and better aligned with OR tracts after contextual processing. In approach A, the Meyer's loop is better preserved when applying both sharpening and enhancement (bottom) than applying sharpening alone (middle). Furthermore, it has less false positives than scoring on initial data, though no critical threshold exists. Approach B uses sharpening of the diffusion profiles by erosion which is clearly beneficial for subsequent enhancement: This approach detects the OR best and sensitivity is further increased after enhancement, which is reflected by a relatively high critical threshold (30%) without false positives. Erosion followed by linear enhancement improves the reconstruction substantially.

**Figure 10:**
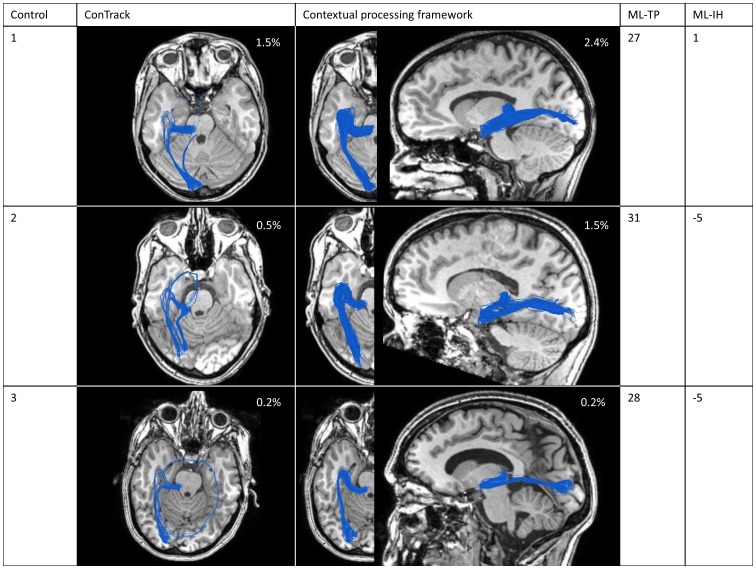
Reconstruction of the optic radiation for the three healthy controls. Comparing the ConTrack method (left) with our method (right), using the result of approach B with 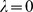 (only based on the data). The percentage of remaining pathways is indicated. The Meyer's loop to temporal pole (ML-TP) and Meyer's loop to inferior horn (ML-IH) distance are shown in columns 4 and 5.

For the remaining healthy controls, the OR including Meyer's loop could also be successfully reconstructed using approach B. The results of scoring on the whole data set of 100 000 fibers can be seen in Fig. 10, where we compared the ConTrack method (left), to our method (right). The ConTrack measure leaves false positives that have to be removed manually by visual inspection to obtain a plausible reconstruction, and no critical threshold exists for any of the three subjects. The ConTrack scoring measure appears to have a weaker discriminating power between anatomically plausible and implausible pathways. When using our framework, critical thresholds do exist. The resulting plausible reconstructions including the Meyer's loop are shown for all three cases.

For quantitative evaluation, we measured the ML-TP distance to be 27, 31 and 28 mm, and ML-IH distance to be 1, −5 and −5 mm for the three subjects, respectively. This is in good agreement with the ranges given in [25], where they reported 22 to 37 mm and −5 to 10 mm for ML-TP and ML-IH, respectively.

### Patients with a brain lesion

For the reconstruction of the OR in patients with disrupted anatomy due to a lesion, approach B was used.

**Figure 11:**
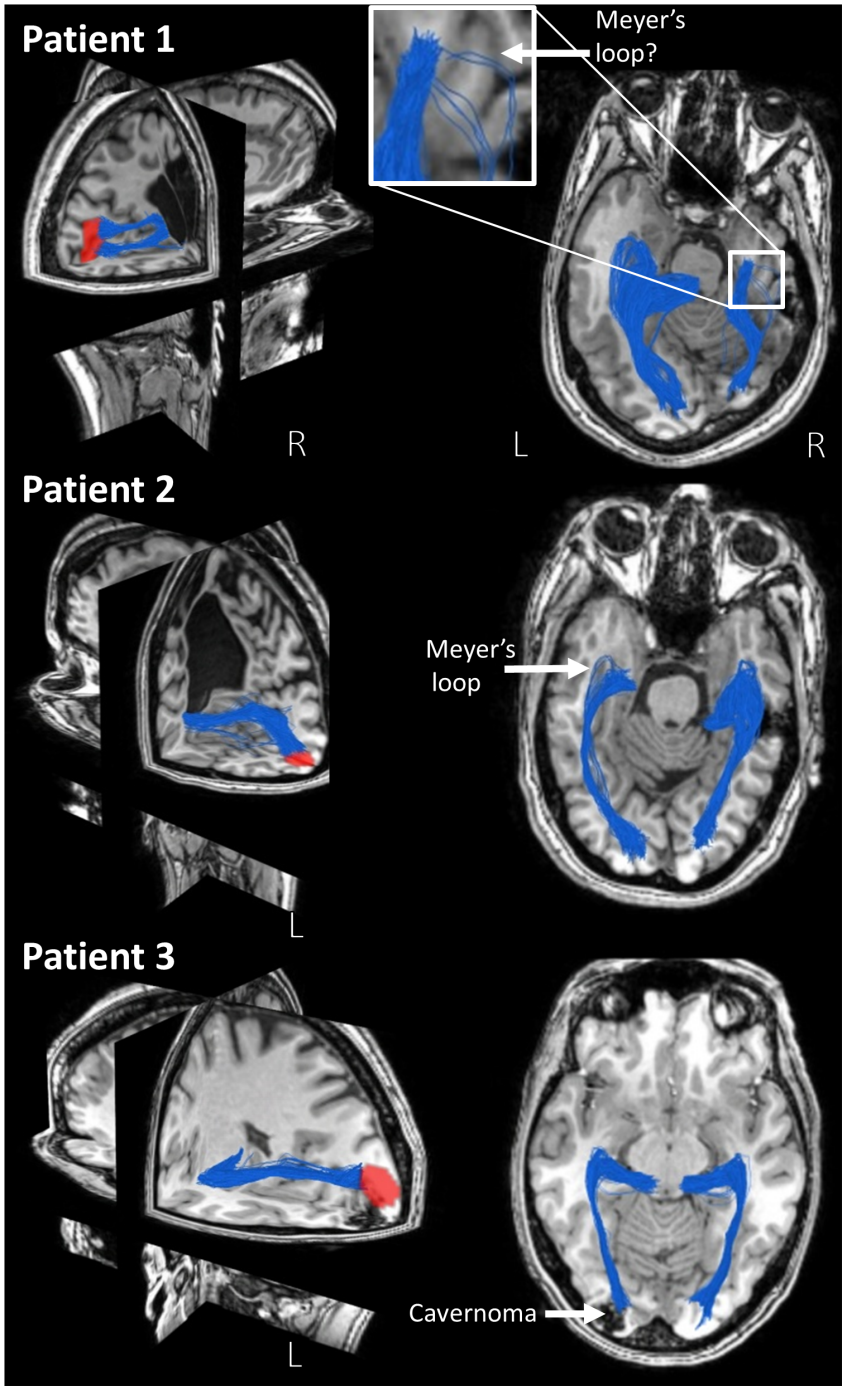
fMRI and tractography results of three patients with an epileptogenic lesion. In the 3D view, the fMRI-activation upon contralateral visual stimulation is visualized in the pathological hemisphere, together with the reconstruction of the OR which was guided by fMRI. In the axial view, the reconstruction of the OR both on the pathological and healthy side can be appreciated. Patient 1 has a cyst in the right hemisphere, patient 2 in the left hemisphere, and patient 3 has a cavernoma in the occipital area.

For patient 1, visual stimulation with a right half field showed a normal distribution of significant BOLD regions in the healthy left hemisphere. For stimulation with the left half field, BOLD activity became apparent in both the right pathologic hemisphere (displayed in Fig. 11 top left) and the left hemisphere (not displayed). Perimetry results showed that the patient only suffered from a lower quadrantanopsia, suggesting that the Meyer's loop should still be intact. Using the significant BOLD region in the pathologic hemisphere as seedpoint, pathways that possibly represent the remainder of the OR including Meyer's loop in the pathological hemisphere could be reconstructed. For the scoring we set 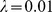 and 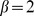 to slightly punish length and curvature, as the pathological hemisphere is much smaller. Since visual function is still located in this hemisphere, hemispherectomy would likely lead to additional visual loss.

In patient 2, normal response was found for both left and right half field stimulation in the contralateral hemisphere (Fig. 11 middle shows activation in the left pathological hemisphere). The patient did not have a visual field deficit despite the large malformations, which was confirmed by the fMRI results that indicated a normal representation of the BOLD regions in the primary visual areas for both left and right half field stimulation. The highest scoring pathways are visualized, which likely represent the OR including Meyer's loop in the pathological hemisphere. In this patient, hemispherectomy would also result in visual loss.

For patient 3, the fMRI results imply that the primary visual cortex is displaced due to the cavernoma. Tractography from the significant BOLD area is visualized in the bottom of Fig. 11, which shows a plausible reconstruction of the OR compared to the healthy hemisphere, providing support for the correctness of the fMRI results. The apparent relocation of visual function to the margins of the space-occupying malformation makes that this patient is still under consideration as a candidate for resective surgery.

## Discussion

In this work, we have evaluated contextual processing on 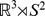 in a clinically relevant application. First, we will discuss the contextual processing on 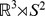. Subsequently, the remainder of the evaluation framework is discussed, including our choice of using DTI data and probabilistic fibers tracked on DTI data as input, the scoring measure, and challenges in the combination of fMRI and DTI. The importance of reliable reconstruction of the OR in clinical practice and the contribution of our framework is outlined afterwards. Finally, directions are given for future work.

### Contextual processing on 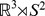 in clinical applications

The contextual processing techniques on 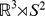 used in this paper combine spatial and orientational information, which has been shown to be beneficial for sharpening and enhancement of diffusion profiles [4], [11], [14], [17], [19]. They are well-posed and formalized in a stable and novel mathematical PDE framework, which allows for generalization and extension of well-known image processing techniques to the domain of dMRI data in future work. The stability of the approach comes along with the obvious stability of the left-invariant PDE evolutions on 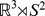. The stochastic interpretation of these evolutions come at hand in the physical interpretation of the contextual processing and the semigroup property missing in other choices [17] of alignment kernels on 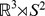.

In this work, enhancement techniques were evaluated on fiber tracking in a clinical setting. Contextual processing obviously has value in clinical applications, as was shown for the reconstruction of the OR in patients with severe pathological malformation (see Fig. 11). By incorporating it in a clinical work-flow, we were able to evaluate this technique in a challenging practical problem: the reconstruction of complex, but clinically eloquent, fiber pathways such as the OR. Contextual processing of one data set takes around 10 minutes in total (for both enhancement and sharpening). Possible improvements could include a reduction of computation time, and increased data adaptivity. To reduce computation time, we propose to combine these two steps in a single evolution, and to use parallel computing. It is useful to increase data-adaptivity by not relying on a single Green's function as a model for propagation of oriented particles as we currently do, but instead adapt the PDE's and Green's function to the data by a statistical training approach.

### Evaluation framework

In order to compare our techniques to clinically used approaches for OR reconstruction such as ConTrack [33], we have used the same input, which is DTI data and the fibers tracked on DTI data resulting from the ConTrack sampling step. Here arise two potential limitations of the evaluation of our method, which deserve discussion. Firstly, the ConTrack sampling step for obtaining a distribution of probabilistic pathways is based on a heuristic approach for characterization of the uncertainty orientation density function at each location, combined with a bootstrapping approach. It does take into account the variability due to noise and modeling errors in this way, but the relationship between the underlying microstructure and the uncertainty function is ad hoc. The distribution of fibers that is used as input for our framework is still based on ConTracks probabilistic sampling. Any other probabilistic fiber tractography algorithm could be used. In future work, the probabilistic tracking will also be incorporated in our framework. Secondly, our contextual processing framework is applied on input data that uses the diffusion tensor model, which is known to be incorrect in areas of crossing fibers. However, the motivation of using DTI data is two-fold. To the best of our knowledge, OR reconstruction (including Meyer's loop) methods that are used in clinical applications are still based on DTI data. To be able to compare our framework to such methods that can reconstruct Meyer's loop (e.g. ConTrack), we used the same input data. Furthermore, there were indications that enhancement based on DTI gives a similar information gain in areas of crossing fibers as some HARDI modeling techniques [14]. This does not mean that only DTI data can be used within the framework. Note that our framework needs an ODF field as input and can thus be generically applied. Further research is needed on how to incorporate the amount of diffusion in the term 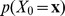 in Eq. (4). The trace for example, is a measure that is more easily generalized to higher order tensors. The fiber distribution function resulting from spherical deconvolution approaches [61]–[63] is a function that is not normalized per voxel, and could directly be used as input 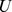. Preliminary results show that this approach is very promising, and future work will be directed to this.

The extraction of the most reliable pathways from the large amount of pathways was done by scoring and subsequent thresholding. We found that the ConTrack scoring measure had a weaker discriminating power between anatomically plausible and implausible fibers compared to our framework. In most cases, we could rely on the data solely (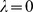). In the case of abnormal brain morphology, using the parameters with clear geometric interpretation allowed us to still obtain a plausible reconstruction. In future work, optimal parameter settings via statistical adaptation will be investigated for other clinically eloquent pathways to widen application scope.

### Combination of fMRI and dMRI

The combination of fMRI and dMRI allows for incorporation of both spatial functional and structural information. The visual stimuli that we use have shown to specifically activate the primary visual cortex [53]. The use of functional activation maps resulting from fMRI for seedpoint location of DW tractography faces some challenges [42], as the BOLD response is mainly located in the grey matter, which has lower anisotropy. The ConTrack sampling step can deal with these areas of low anisotropy due to its probabilistic nature. Furthermore, it is quite challenging to find a standardized method that can extract the seed regions for fiber tracking from the BOLD activity maps. These maps often show more than one activated area, but we found that the appropriate threshold on the activity map was rather constant between individuals. Despite the fact that our solution to this problem included manual extraction of the seed point from the activity maps, these maps were extremely useful in localizing the visual cortex in patients with disrupted anatomy due to a lesion. For these patients, it appeared that the specificity of the reconstructed path of the OR highly relies on proper localization of the seed point based on fMRI.

### Importance of reliable reconstruction of the OR in clinical practice

Reliable delineation of complex white matter pathways such as the OR is important in neurosurgical applications to spare function, in this case visual function. The patients in this study were evaluated as candidates for epilepsy surgery in order to render them seizure free, without disrupting visual function. Assessment of visual function in the patients included in this paper was rather challenging due to the complex brain morphology and its associated symptoms. fMRI and perimetry results were sometimes hard to interpret in these patients, which raises questions on the reliability. Patient 1, for example, had a lower quadrantanopsia, which is quite extraordinary. fMRI results confirmed that there was visual function left in the pathological hemisphere, but also in the ipsilateral healthy hemisphere. This raises the question whether the BOLD response in the pathological hemisphere indicated true activity. dMRI gave the opportunity to underpin this hypothesis by looking at plausible pathway reconstructions originating from the activated area, which indicated that visual function is still located in this hemisphere. Furthermore, while fMRI GE-EPI images and their corresponding activity maps are vulnerable to nearby vascular malformations (like in patient 3), SE-EPI sequences are less affected by this problem and tractography gives plausible results around vascular malformations [64], [65]. It was found that tractography is a useful technique in localizing the OR and evaluating surgical risk. For patient 3 included in this study, the tractography results support the fMRI findings, since the reconstruction terminates in the activated area and looks plausible when comparing it to the healthy hemisphere. In conclusion, the combination of fMRI and reliable tractography on dMRI data provides information on whether resection of the lesion might be possible without disruption of visual function. Thus, the fMRI and DTI results contribute to the clinical question whether these patients can (still) be considered candidates for epilepsy surgery.

The analysis framework as described can also be useful for the reconstruction of the OR in order to prevent a visual field deficit in case of an anterior temporal lobe resection [66], [67]. Temporal Lobe Epilepsy is characterized by seizures originating in or primarily involving temporal lobe structures such as the hippocampus, amygdala, and parahippocampal gyrus. In standard anterior temporal lobe resection, 4.0 to 6.5 cm of the anterior temporal lobe and portions of the medial structures are resected [68], after which approximately 60 to 80% of the patients become seizure free [69]. The most common complication (arising in up to 100% of the cases) is a visual field deficit in the upper quadrant of the visual field [21]–[23]. This is due to disruption of the anterior part of the OR (Meyer's loop), since it is often located in the resection area.

### Limitations and future work

Before the methods as presented in this paper can become established in clinical routine, improvements in methodology and better understanding of the interaction between pathology and pathway reconstruction are needed. Validation of reconstructed paths with dMRI is always a major point of discussion, and of particular importance when using it in neurosurgical applications. The reconstructions obtained with the method presented in this paper can be used in future (randomized) studies that aim to relate resection size in temporal lobe epilepsy surgery to visual field deficit, or more specifically, to relate the amount of resection within the OR to the visual loss. These studies are currently limited by difficulties in reliable delineation of the anterior extent of Meyer's loop and by the lack of uniform methods to quantify the size and shape of the visual loss [70]. The benefit of such quantitative studies with proper methodology is two-fold: the reconstruction including Meyer's loop can be validated, and more insight into the functional organization of the distinct bundles of the OR (posterior and central bundle and Meyer's loop [29], [71]) can be obtained. Furthermore, the consequence of EPI distortions for OR reconstruction and the ways to correct for these distortions have to be investigated, as the OR (and Meyer's loop) is close to air-tissue interfaces such as the sinuses near the temporal lobe. EPI is the method of choice for dMRI and fMRI since it is a fast imaging technique, but the resulting images are inherently distorted due to the heterogeneity of brain tissue and its corresponding susceptibility differences. Especially at air-tissue interfaces, geometric distortions can be of significant magnitude, which influences tractography results [72]. Tracts can appear to be less symmetric or terminate in different sulci in susceptibility distorted images.

## Conclusions

In this paper, we have evaluated contextual processing on 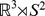 in a clinically relevant application: the reconstruction of the OR for temporal lobe surgery. The evaluation was done by assigning a score to each pathway based on its fit to the underlying data and its geometry, and looking at the anatomical plausibility of the highest scoring pathways. When applying sharpening and enhancement, sensitivity and specificity of the extraction of anatomically plausible pathways are greatly improved. Our methods are able to extract a larger amount of anatomically plausible pathways with 100% specificity, compared to another well-established method. Therefore, contextual processing on 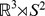 is beneficial for investigating the structure of the brain in a more reliable way. In three patients with disrupted anatomy due to a lesion we were able to obtain a plausible reconstruction of the optic radiation by integrating functional information from fMRI. Our results demonstrate that the framework contributes to answering the clinical question whether a patient can have resective surgery, while sparing visual function.

## Supporting Information

Appendix S1 **Abbreviation table.**
(PDF)

Appendix S2 **Enhancement and sharpening.**
(PDF)

Appendix S3 **Scoring measure.**
(PDF)
