# Supplementary material for: Evaluating Contextual Processing in Diffusion MRI: Application to Optic Radiation Reconstruction for Epilepsy Surgery
Source: PLoS One. 2014 Jul 31;9(7):e101524. doi: 10.1371/journal.pone.0101524 (PMC4117467; doi:10.1371/journal.pone.0101524)
Supplement: Appendix S1 — Abbreviation table. (PDF) [file pone.0101524.s001.pdf]

Appendix S1: Abbreviation table

$SE(n)$	n-dimensional special Euclidean motion group
$\mathbb{R}^n$	n-dimensional position space
$SO(n)$	n-dimensional rotation group
S^n	n-dimensional sphere
$[D^{ij}] \in \mathbb{R}^{5\times 5}$	Components of diffusion matrix for diffusion processes on $\mathbb{R}^3 \rtimes S^2$
$[G^{ij}] \in \mathbb{R}^{5\times 5}$	Components of left-invariant inverse metric tensor on $\mathbb{R}^3 \rtimes S^2$ for erosion processes on $\mathbb{R}^3 \rtimes S^2$
$\mathbf{n} = \mathbf{n}(\tilde{\beta}, \tilde{\gamma}) \in S^2$	Orientation
$R_{\mathbf{n}}$	Rotation matrix which maps $\mathbf{e}_z$ to $\mathbf{n}$
$\{\mathcal{A}_i\}_{i=1}^5$	Local frame of reference
$(\mathbf{x}, \mathbf{n}) \mapsto U(\mathbf{x}, \mathbf{n})$	Function U on $\mathbb{R}^3 \rtimes S^2$
$(\mathbf{x}, \mathbf{n}) \mapsto W(\mathbf{x}, \mathbf{n}, t)$	$W(\cdot, t)$ on $\mathbb{R}^3 \rtimes S^2$ at time $t > 0$
$\{N_t\}$	Random variable denoting the direction in S^2
$\{X_t\}$	Random variable denoting the position in $\mathbb{R}^3$
BOLD	Blood-oxygen-level-dependent
dMRI	Diffusion MRI
DTI	Diffusion tensor imaging
DWI	Diffusion weighted imaging
EPI	Echo-planar imaging
fMRI	Functional MRI
FOV	Field of view
HARDI	High angular resolution diffusion imaging
LGN	Lateral geniculate nucleus
NSA	Number of signal averages
ODF	Orientation density function
OR	Optic radiation
PDF	Probability density function
TE	Echo time
TR	Repetition time
