# Supplementary material for: Evaluating Contextual Processing in Diffusion MRI: Application to Optic Radiation Reconstruction for Epilepsy Surgery
Source: PLoS One. 2014 Jul 31;9(7):e101524. doi: 10.1371/journal.pone.0101524 (PMC4117467; doi:10.1371/journal.pone.0101524)
Supplement: Appendix S2 — Enhancement and sharpening. (PDF) [file pone.0101524.s002.pdf]

Appendix S2: Enhancement and sharpening

1 Moving frame of reference

Expressions for the moving frame of reference without using Euler angles [1, 2]:

$$\begin{aligned}
\mathcal{A}_1 U(\mathbf{x},\mathbf{n}) &= \lim_{h\to 0} \frac{U(\mathbf{x}+hR_{\mathbf{n}}\mathbf{e}_x,\mathbf{n})-U(\mathbf{x},\mathbf{n})}{h}\\
\mathcal{A}_2 U(\mathbf{x},\mathbf{n}) &= \lim_{h\to 0} \frac{U(\mathbf{x}+hR_{\mathbf{n}}\mathbf{e}_y,\mathbf{n})-U(\mathbf{x},\mathbf{n})}{h}\\
\mathcal{A}_3 U(\mathbf{x},\mathbf{n}) &= \lim_{h\to 0} \frac{U(\mathbf{x}+hR_{\mathbf{n}}\mathbf{e}_z,\mathbf{n})-U(\mathbf{x},\mathbf{n})}{h}\\
\mathcal{A}_4 U(\mathbf{x},\mathbf{n}) &= \lim_{h_a\to 0} \frac{U(\mathbf{x}+R_{\mathbf{n}}R^{\mathbf{e}_x}_{h_a}\mathbf{e}_z,\mathbf{n})-U(\mathbf{x},\mathbf{n})}{h_a}\\
\mathcal{A}_5 U(\mathbf{x},\mathbf{n}) &= \lim_{h_a\to 0} \frac{U(\mathbf{x}+R_{\mathbf{n}}R^{\mathbf{e}_y}_{h_a}\mathbf{e}_z,\mathbf{n})-U(\mathbf{x},\mathbf{n})}{h_a}.
\end{aligned} \tag{1}$$

Where $R_{\mathbf{n}}$ is any rotation which rotates $\mathbf{e}_z$ to $\mathbf{n}$ via $\mathbf{n} = R_{\mathbf{n}}\mathbf{e}_z$, and $R^{\mathbf{e}_i}_{h_a}$ denotes a counterclockwise rotation of angle h_a around $\mathbf{e}_i$. Although the choice of $R_{\mathbf{n}}$ does matter in the derivatives in Eq. 1, in the combinations used in the enhancement and sharpening operations it does not.

2 Convolution for linear contour enhancement

The following analytical approximation for the convolution kernel for linear contour enhancement in $\mathbb{R}^3 \rtimes S^2$ can be derived from $\mathbb{R}^2 \rtimes S^1$ kernels (for further details see [1]):

$$p^{\mathbb{R}^3\rtimes S^2}_{D^{33},D^{44},t}(x,y,z,\mathbf{n}(\tilde{\beta},\tilde{\gamma})) = p^{\mathbb{R}^2\rtimes S^1}_{D^{33},D^{44},t}(z/2,x,\tilde{\beta})\,p^{\mathbb{R}^2\rtimes S^1}_{D^{33},D^{44},t}(z/2,-y,\tilde{\gamma}) \tag{2}$$

$$\text{with } p^{\mathbb{R}^2\rtimes S^1}_{D^{33},D^{44},t}(x,y,\theta) = \tag{3}$$

$$\begin{cases}
\frac{1}{4\pi t^2 D^{33}D^{44}} \exp\left(-\frac{1}{4t}\sqrt{\left(\frac{\theta^2}{D^{44}}+\frac{\theta^2(y-(-x\sin\theta+y\cos\theta))^2}{4(1-\cos\theta)^2 D^{33}}\right)^2+\frac{1}{D^{33}D^{44}}\left|\frac{\theta((x\cos\theta+y\sin\theta)-x)}{2(1-\cos\theta)}\right|^2}\right), & \text{if } \theta\neq 0\\
\frac{1}{4\pi t^2 D^{33}D^{44}} \exp\left(-\frac{1}{4t}\sqrt{\left(\frac{x^2}{D^{33}}\right)^2+\frac{y^2}{D^{33}D^{44}}}\right), & \text{if } \theta = 0.
\end{cases} \tag{4}$$

Convolution in $\mathbb{R}^3 \rtimes S^2$ is then obtained by

$$\begin{aligned}
W(\mathbf{x},\mathbf{n},t) &= (p^{\mathbb{R}^3\rtimes S^2}_{D^{33},D^{44},t} *_{\mathbb{R}^3\rtimes S^2} U)(\mathbf{x},\mathbf{n})\\
&= \int_{\mathbb{R}^3}\int_{S^2} p^{\mathbb{R}^3\rtimes S^2}_{D^{33},D^{44},t}(R^T_{\mathbf{n}'}(\mathbf{x}-\mathbf{x}'),R^T_{\mathbf{n}'}\mathbf{n})U(\mathbf{x}',\mathbf{n}')d\sigma(\mathbf{n}')d\mathbf{x}',
\end{aligned} \tag{5}$$

in which the kernel is both rotated and translated, and multiplied with the image.

3 Finite differences

Implementation of forward finite difference schemes to approximate Eq. (5) in the manuscript for linear contour enhancement is done as follows [2]:

$$\begin{cases}
W(\mathbf{x},\mathbf{n},t+\Delta t) = W(\mathbf{x},\mathbf{n},t) + \Delta t\left(D^{33}(\mathcal{A}^c_3)^2 + D^{44}\left((\mathcal{A}^c_4)^2+(\mathcal{A}^c_5)^2\right)W\right)(\mathbf{x},\mathbf{n},t)\\
W(\mathbf{x},\mathbf{n},0) = U(\mathbf{x},\mathbf{n}),
\end{cases} \tag{6}$$

with

$$((\mathcal{A}_1^c)^2U)(\mathbf{y},\mathbf{n}) = \frac{U(\mathbf{x}+hR_{\mathbf{n}}\mathbf{e}_x,\mathbf{n}) - 2\,U(\mathbf{x},\mathbf{n}) + U(\mathbf{x}-hR_{\mathbf{n}}\mathbf{e}_x,\mathbf{n})}{h^2} \tag{7}$$

$$((\mathcal{A}_2^c)^2U)(\mathbf{y},\mathbf{n}) = \frac{U(\mathbf{x}+hR_{\mathbf{n}}\mathbf{e}_y,\mathbf{n}) - 2\,U(\mathbf{x},\mathbf{n}) + U(\mathbf{x}-hR_{\mathbf{n}}\mathbf{e}_y,\mathbf{n})}{h^2} \tag{8}$$

$$((\mathcal{A}_3^c)^2U)(\mathbf{y},\mathbf{n}) = \frac{U(\mathbf{x}+hR_{\mathbf{n}}\mathbf{e}_z,\mathbf{n}) - 2\,U(\mathbf{x},\mathbf{n}) + U(\mathbf{x}-hR_{\mathbf{n}}\mathbf{e}_z,\mathbf{n})}{h^2} \tag{9}$$

$$((\mathcal{A}_4^c)^2U)(\mathbf{y},\mathbf{n}) = \frac{U(\mathbf{x}\,,\,R_{\mathbf{n}}\,R_{h_a}^{\mathbf{e}_x}\,\mathbf{e}_z) - 2\,U(\mathbf{x},\mathbf{n}) + U(\mathbf{x}\,,\,R_{\mathbf{n}}\,R_{-h_a}^{\mathbf{e}_x}\,\mathbf{e}_z)}{{h_a}^2} \tag{10}$$

$$((\mathcal{A}_5^c)^2U)(\mathbf{y},\mathbf{n}) = \frac{U(\mathbf{x}\,,\,R_{\mathbf{n}}\,R_{h_a}^{\mathbf{e}_y}\,\mathbf{e}_z) - 2\,U(\mathbf{x},\mathbf{n}) + U(\mathbf{x}\,,\,R_{\mathbf{n}}\,R_{-h_a}^{\mathbf{e}_y}\,\mathbf{e}_z)}{{h_a}^2}. \tag{11}$$

Here, the superscript c denotes that we use centered finite differences (in this case of second order). For more information on this numerical implementation and its stability, see [2]. For the finite difference implementation of erosions, [3] and [4] can be consulted.

References

1. Duits R, Franken E (2011) Left-Invariant Diffusions on the Space of Positions and Orientations and their Application to Crossing-Preserving Smoothing of HARDI images. Int J Comput Vis 92: 231–264.

2. Creusen E, Duits R, Vilanova A, Florack L (2013) Numerical Schemes for Linear and Non-Linear Enhancement of DW-MRI. Numer Math Theory Methods and Appl 6: 138–168.

3. Duits R, Dela Haije T, Creusen E, Ghosh A (2013) Morphological and Linear Scale Spaces for Fiber Enhancement in DW-MRI. J Math Imaging Vis 46: 326–368.

4. Dela Haije TCJ, Duits R, Tax CMW (2014) Sharpening Fibers in Diffusion Weighted MRI via Erosion. In: Westin C, Vilanova A, Burgeth B, editors, Visualization and Processing of Tensors and Higher Order Descriptors for Multi-Valued Data, Springer. (in press).
