# Supplementary material for: Evaluating Contextual Processing in Diffusion MRI: Application to Optic Radiation Reconstruction for Epilepsy Surgery
Source: PLoS One. 2014 Jul 31;9(7):e101524. doi: 10.1371/journal.pone.0101524 (PMC4117467; doi:10.1371/journal.pone.0101524)
Supplement: Appendix S3 — Scoring measure. (PDF) [file pone.0101524.s003.pdf]

Appendix S3: Scoring measure

The new scoring method is based on a sub-Riemannian distance on $\mathbb{R}^3 \rtimes S^2$ and inspired by sub-Riemannian geodesics modeling the association fields for alignment in the primary visual cortex [1]. Here it is derived from Bayesian interference, and should represent how likely the pathway $\gamma(s) = (\mathbf{x}(s), \mathbf{n}(s))$ is given the data $U : \mathbb{R}^3 \rtimes S^2 \to \mathbb{R}$, which is a distribution on positions and orientations.

$$p(\gamma \mid U) = \frac{p(U \mid \gamma)p(\gamma)}{p(U)}. \tag{1}$$

In this equation, $p(\gamma \mid U)$ is the posterior distribution that has to be calculated, whereas $p(U \mid \gamma)$ is the conditional distribution that will be referred to as the 'data dependent term'. In this term, the underlying diffusion information is taken into account. The density $p(\gamma)$ is the prior distribution which we will refer to as the 'data independent term'. This term can impose restrictions on the geometry of the pathway. To obtain the log likelihood, the logarithm of this equation is taken:

$$\log(p(\gamma \mid U)) = \log(p(U \mid \gamma)) + \log(p(\gamma)) - \log(p(U)). \tag{2}$$

Note that the logarithm is monotonic and has no influence on the ranking of the fibers. In the subsequent subsections we will address each term.

1 Data dependent term $p(U \mid \gamma)$

For the data dependent term, the following is proposed in analogy to [2]:

$$\log(p(U \mid \gamma)) = \frac{1}{L_\gamma} \int_0^{L_\gamma} \log \frac{U(\gamma(s))}{\max U} ds. \tag{3}$$

Here it is assumed that the probability on the whole diffusion profile field given the curve is the same as the probability on the diffusion profile field along the curve given the curve, in other words $p(U \mid \gamma) = p(U|_\gamma|\gamma)$. The multiplication by $\frac{1}{L_\gamma}$ corrects this term for the length of the curve L_γ and naturally averages the data contributions. This term can be seen as the external energy of the curve.

2 Data independent term $p(\gamma)$

For the data independent term we propose:

$$\log(p(\gamma)) = -\lambda \int_0^{L_\gamma} (\kappa^2(s) + \beta^2)^{\frac{1}{2}} ds. \tag{4}$$

Here, $\kappa(s)$ is the curvature of the curve, and β is a parameter that balances between the cost for spatial and angular movements of an oriented particle. We can tune length in comparison to curvature. This term can be seen as the internal energy of the curve. λ is another user defined parameter that can tune the weight of the internal energy with respect to the external energy. This data independent term corresponds to finding the cuspless sub-Riemannian geodesics in $\mathbb{R}^3 \rtimes S^2$.

3 Total scoring

In the ranking of the fibers, the normalization term in Bayes' rule is assumed to be 1 since it does not alter the ranking of the fibers: $p(U) = 1$ and thus $\log(p(U)) = 0$. By substituting equation (3) and (4)

in equation (2) one obtains:

$$\log p(\gamma \mid U) = \frac{1}{L_\gamma} \int_0^{L_\gamma} \log U(\gamma(s)) ds - \int_0^{L_\gamma} \lambda(\kappa^2(s) + \beta^2)^{\frac{1}{2}} ds. \tag{5}$$

References

1. Duits R, Boscain U, Rossi F, Sachkov Y (2014) Association fields via cuspless sub-riemannian geodesics in SE(2). J Math Imaging Vis 49: 384-417.

2. Mumford D (1991) Elastica and Computer Vision. Center for Intelligent Control Systems, Massachusetts Institute of Technology.
